# Supplementary material for: Cold-Atmospheric Plasma Induces Tumor Cell Death in Preclinical In Vivo and In Vitro Models of Human Cholangiocarcinoma
Source: Cancers (Basel). 2020 May 19;12(5):1280. doi: 10.3390/cancers12051280 (PMC7281400; doi:10.3390/cancers12051280)
Supplement: Supplementary file 1 [file cancers-12-01280-s001.pdf]

Supplemental Materials

Cold-Atmospheric Plasma Induces Tumor Cell Death in Preclinical In Vivo and In Vitro Models of Human Cholangiocarcinoma

Javier Vaquero, Florian Judée, Marie Vallette, Henri Decauchy, Ander Arbelaiz, Lynda Aoudjehane, Olivier Scatton, Ester Gonzalez-Sanchez, Fatiha Merabtene, Jérémy Augustin, Chantal Housset, Thierry Dufour and Laura Fouassier

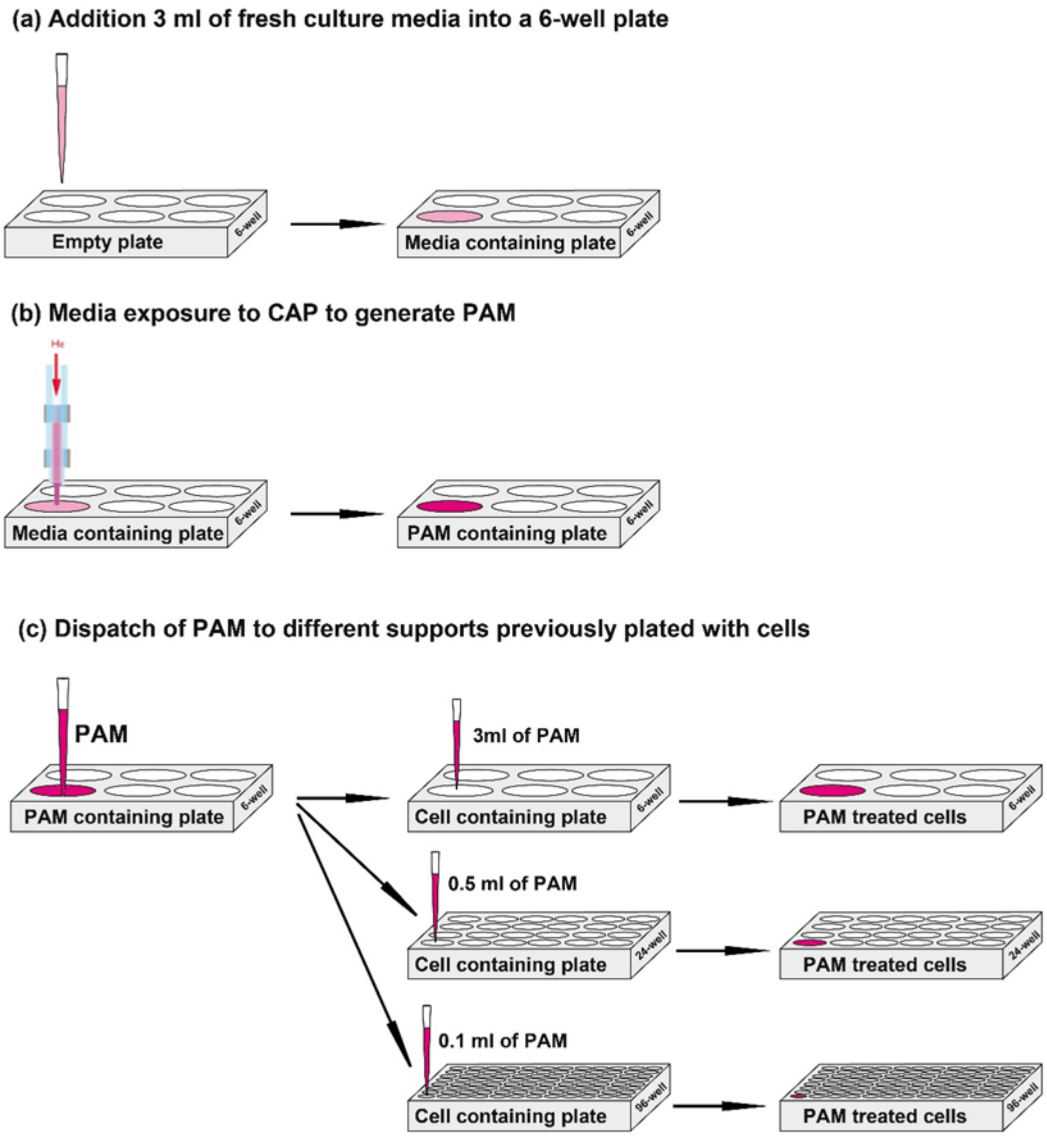

Figure S1. Schematic representation of in vitro treatment of CCA cell lines and hepatocytes with PAM. (**a**) 3 mL of fresh culture media is added into an empty well of a 6-well plate. (**b**) Fresh culture media is treated with CAP during 1, 3, 5 or 10 minutes to generate PAM. (**c**) PAM is collected and dispatched in different plastic supports (i.e., 6-well, 24-well or 96-well plates) previously plated with CCA cell lines or hepatocytes. Then plates are developed by different methods at different time points as explained in the manuscript section Materials and Methods.

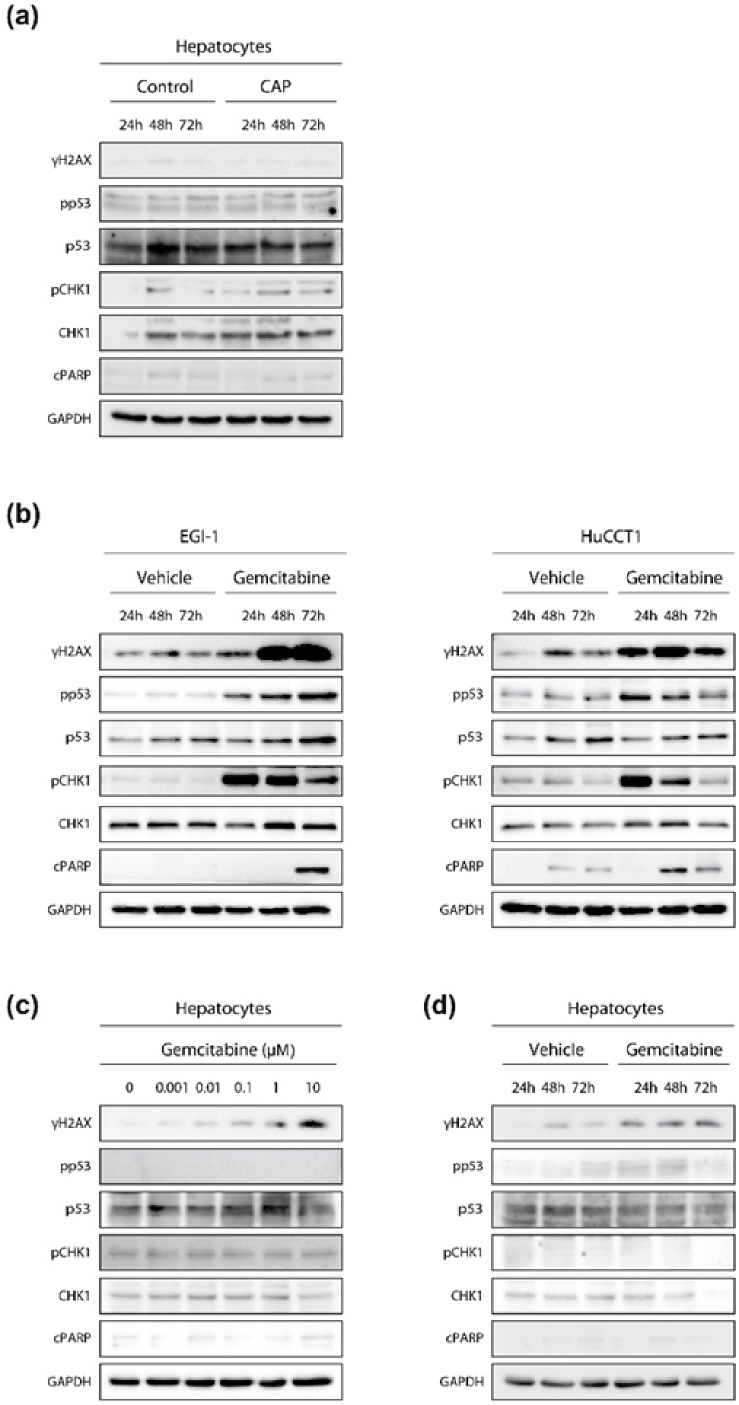

Figure S2. (**a**) Representative images of western blot analysis of cleaved PARP, phosphorylated and total p53, phosphorylated and total CHK1 and phosphorylated H2AX in hepatocytes after 24, 48 and 72 h of exposure to culture medium pretreated with CAP for 3 min (9 kV, 30 kHz, 14%, gap of 7 mm). (**b**,**c**) Representative images of western blot analysis of cleaved PARP, phosphorylated and total p53, phosphorylated and total CHK1 and phosphorylated H2AX in EGI-1 and HuCCT1 after 24h, 48h and 72 h of exposure to 0.1 and 0.01 μM of gemcitabine, respectively. (**d**) Representative images of western blot analysis of cleaved PARP, phosphorylated and total p53, phosphorylated and total CHK1 and phosphorylated H2AX in EGI-1 and HuCCT1 after 72 h of exposure to increasing doses of gemcitabine. (**e**) Representative images of western blot analysis of cleaved PARP, phosphorylated and total p53, phosphorylated and total CHK1 and phosphorylated H2AX in EGI-1 and HuCCT1 after 24, 48 h and 72h of exposure to 10 μM of gemcitabine.

In the following pages we show Figure S3–S10 that contain the western blots performed with 3 different cultures of EGI-1 and HuCCT1 cholangiocarcinoma (CCA) cells exposed to plasma activated medium (PAM), that were quantified to generate the graphs showed in Figure 7 from the manuscript. The densitometry original data from each of the proteins that were quantified (γH2AX, pp53, pCHK1, cPARP and GAPDH) is shown below the corresponding band. We also show the original capture from the PageRuler™ Prestained Protein Ladder (Biorad) taken for each protein and the closer molecular weight marker (PageRuler™ Prestained Protein Ladder, Thermofisher) to each band.

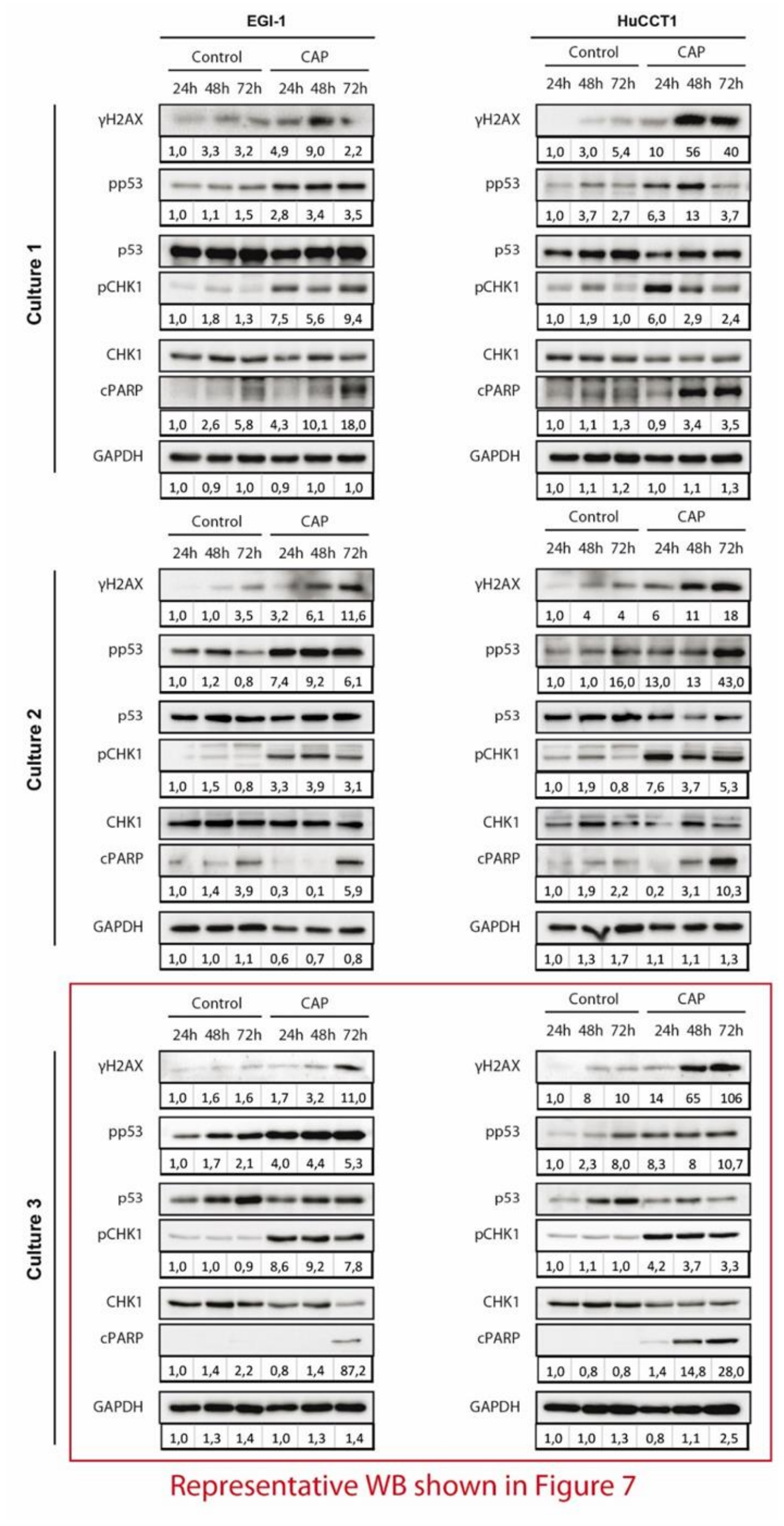
EGI-1
HuCCT1
Control
CAP
24h 48h 72h 24h 48h 72h
Culture 1
Culture 2
Culture 3
γH2AX
pp53
p53
pCHK1
CHK1
cPARP
GAPDH
1,0 3,3 3,2 4,9 9,0 2,2
1,0 1,1 1,5 2,8 3,4 3,5
1,0 1,8 1,3 7,5 5,6 9,4
1,0 2,6 5,8 4,3 10,1 18,0
1,0 0,9 1,0 0,9 1,0 1,0
1,0 3,0 5,4 10 56 40
1,0 3,7 2,7 6,3 13 3,7
1,0 1,9 1,0 6,0 2,9 2,4
1,0 1,1 1,3 0,9 3,4 3,5
1,0 1,1 1,2 1,0 1,1 1,3
1,0 1,0 3,5 3,2 6,1 11,6
1,0 1,2 0,8 7,4 9,2 6,1
1,0 1,5 0,8 3,3 3,9 3,1
1,0 1,4 3,9 0,3 0,1 5,9
1,0 1,0 1,1 0,6 0,7 0,8
1,0 4 4 6 11 18
1,0 1,0 16,0 13,0 13 43,0
1,0 1,9 0,8 7,6 3,7 5,3
1,0 1,9 2,2 0,2 3,1 10,3
1,0 1,3 1,7 1,1 1,1 1,3
1,0 1,6 1,6 1,7 3,2 11,0
1,0 1,7 2,1 4,0 4,4 5,3
1,0 1,0 0,9 8,6 9,2 7,8
1,0 1,4 2,2 0,8 1,4 87,2
1,0 1,3 1,4 1,0 1,3 1,4
1,0 8 10 14 65 106
1,0 2,3 8,0 8,3 8 10,7
1,0 1,1 1,0 4,2 3,7 3,3
1,0 0,8 0,8 1,4 14,8 28,0
1,0 1,0 1,3 0,8 1,1 2,5
Representative WB shown in Figure 7

Figure S3.

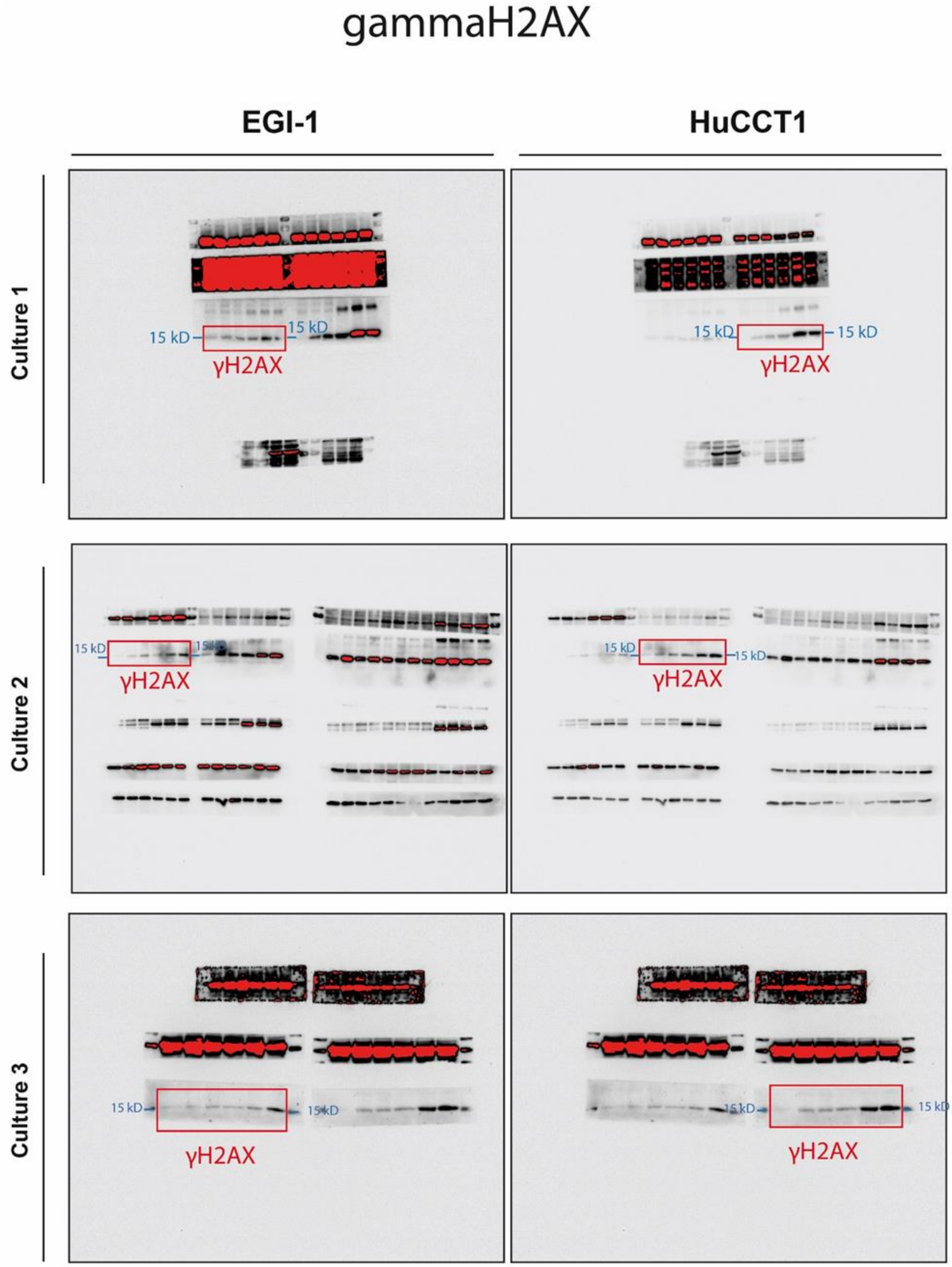
gammaH2AX
EGI-1
HuCCT1
Culture 1
Culture 2
Culture 3
15 kD
γH2AX

Figure S4.

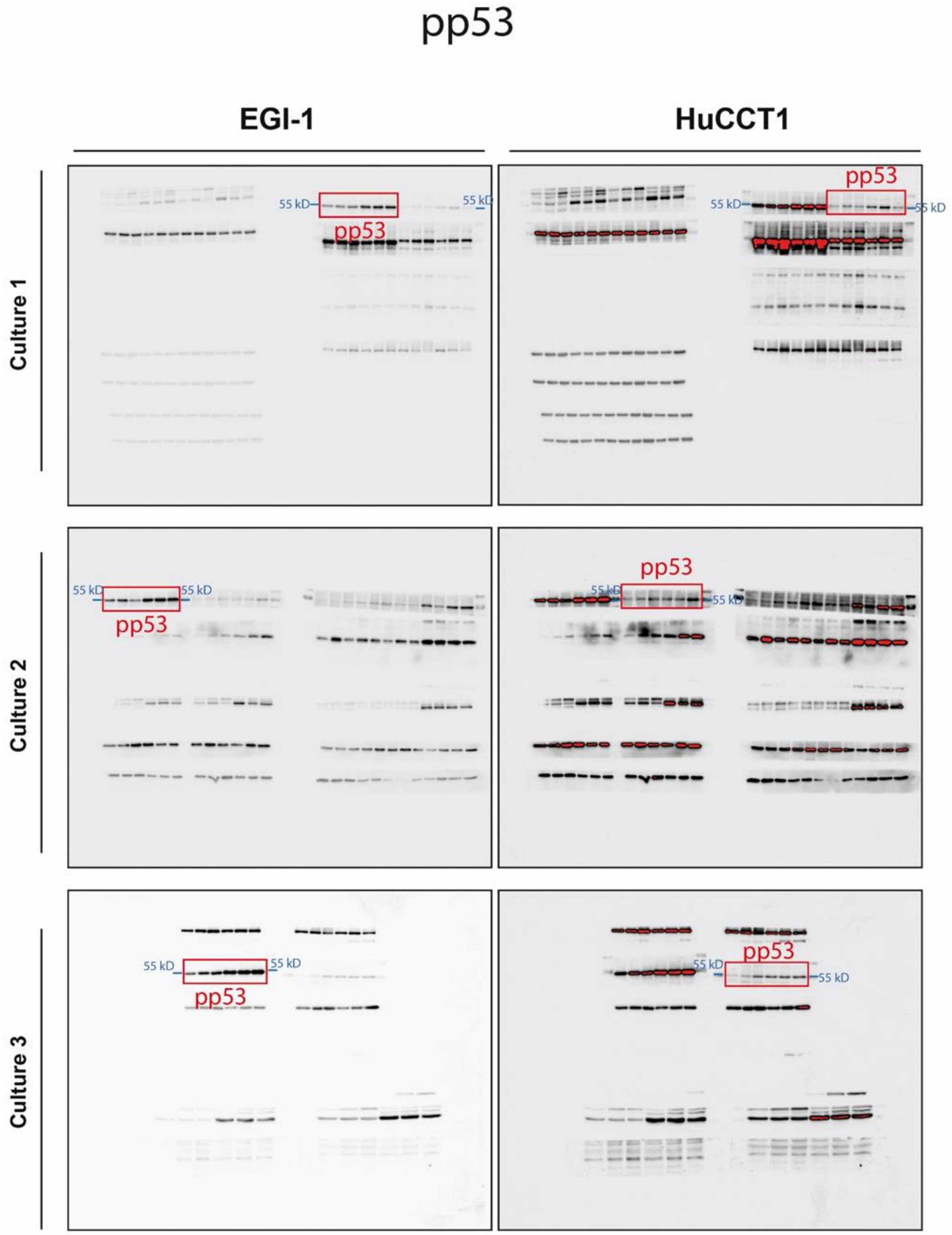

Figure S5.

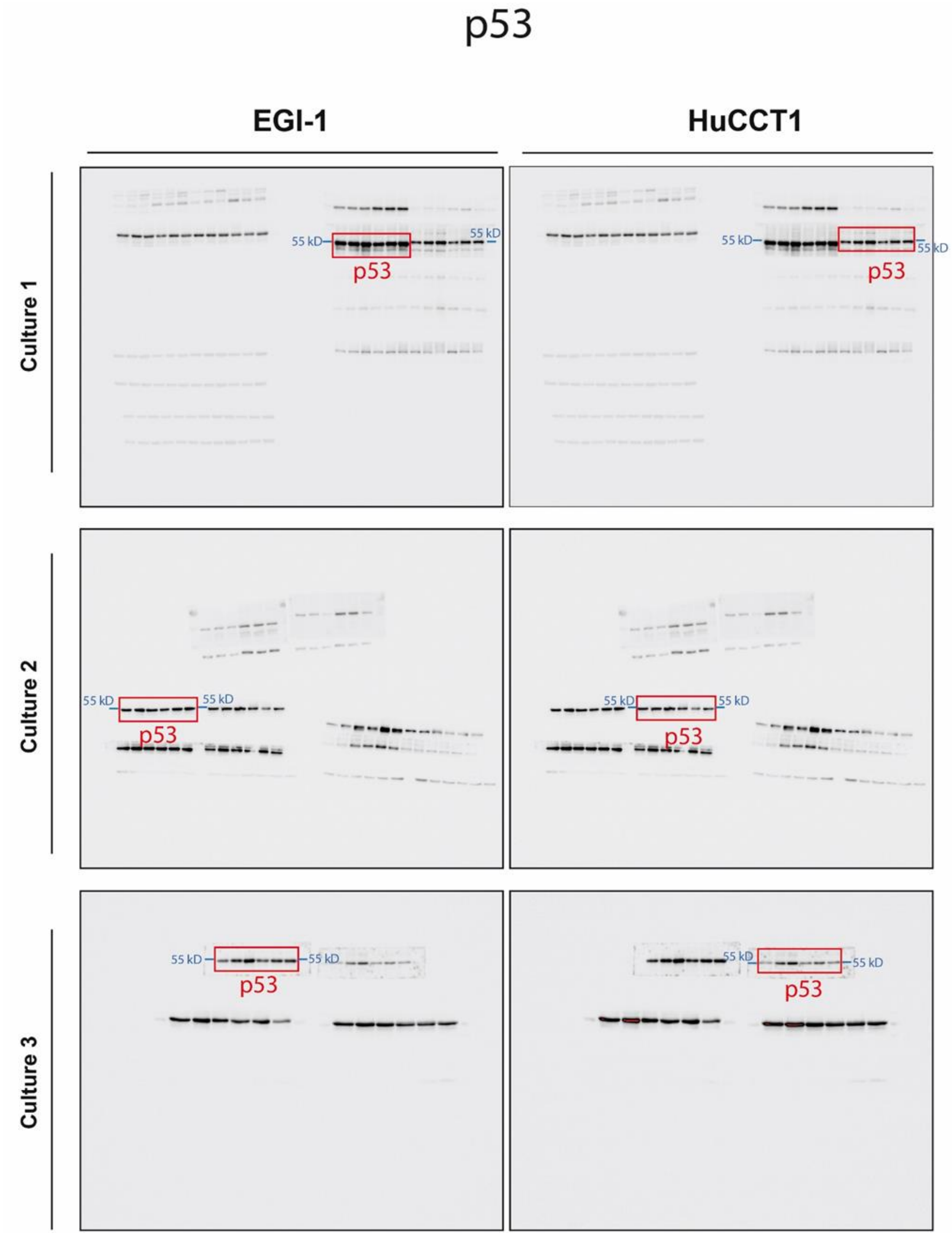

Figure S6.

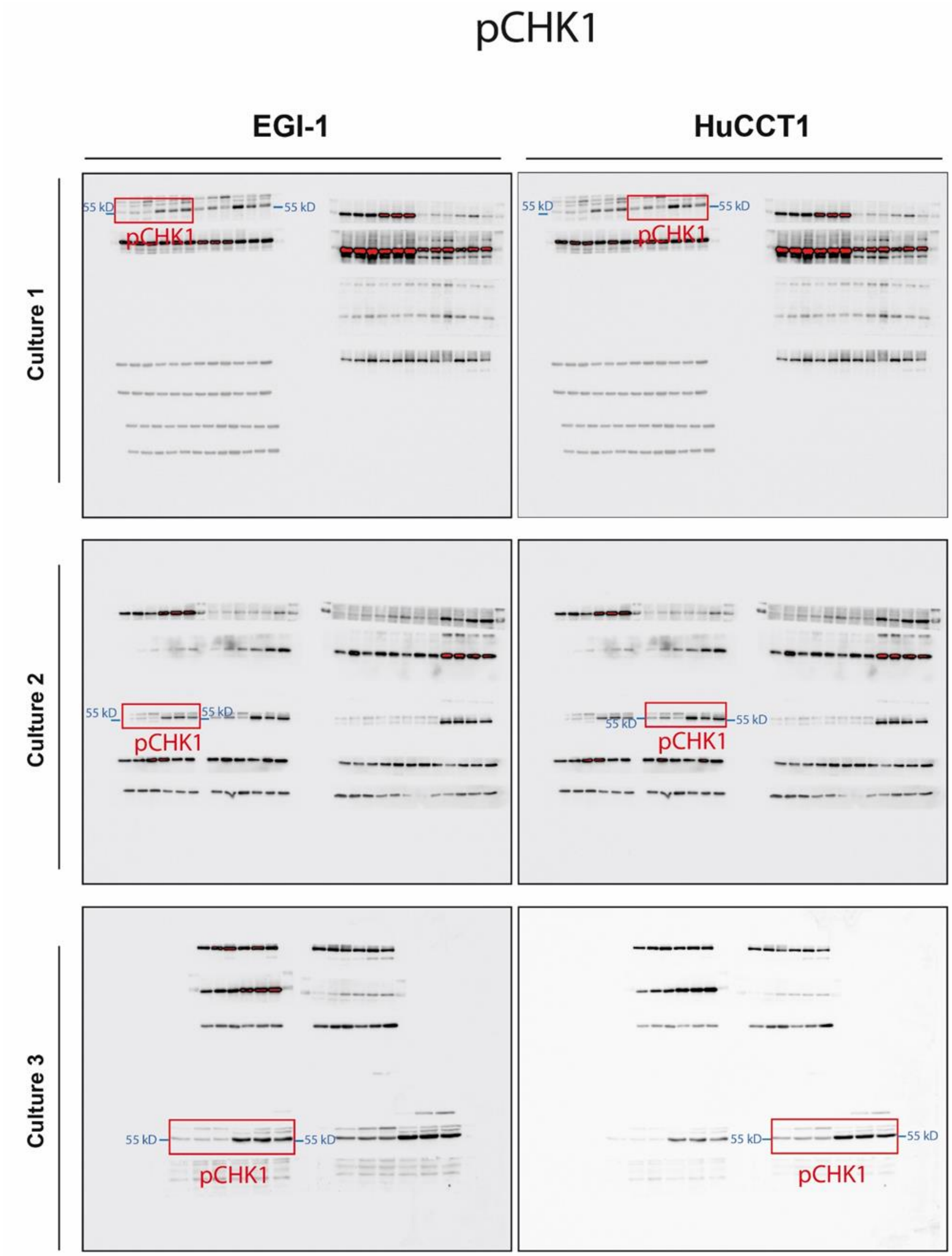

Figure S7.

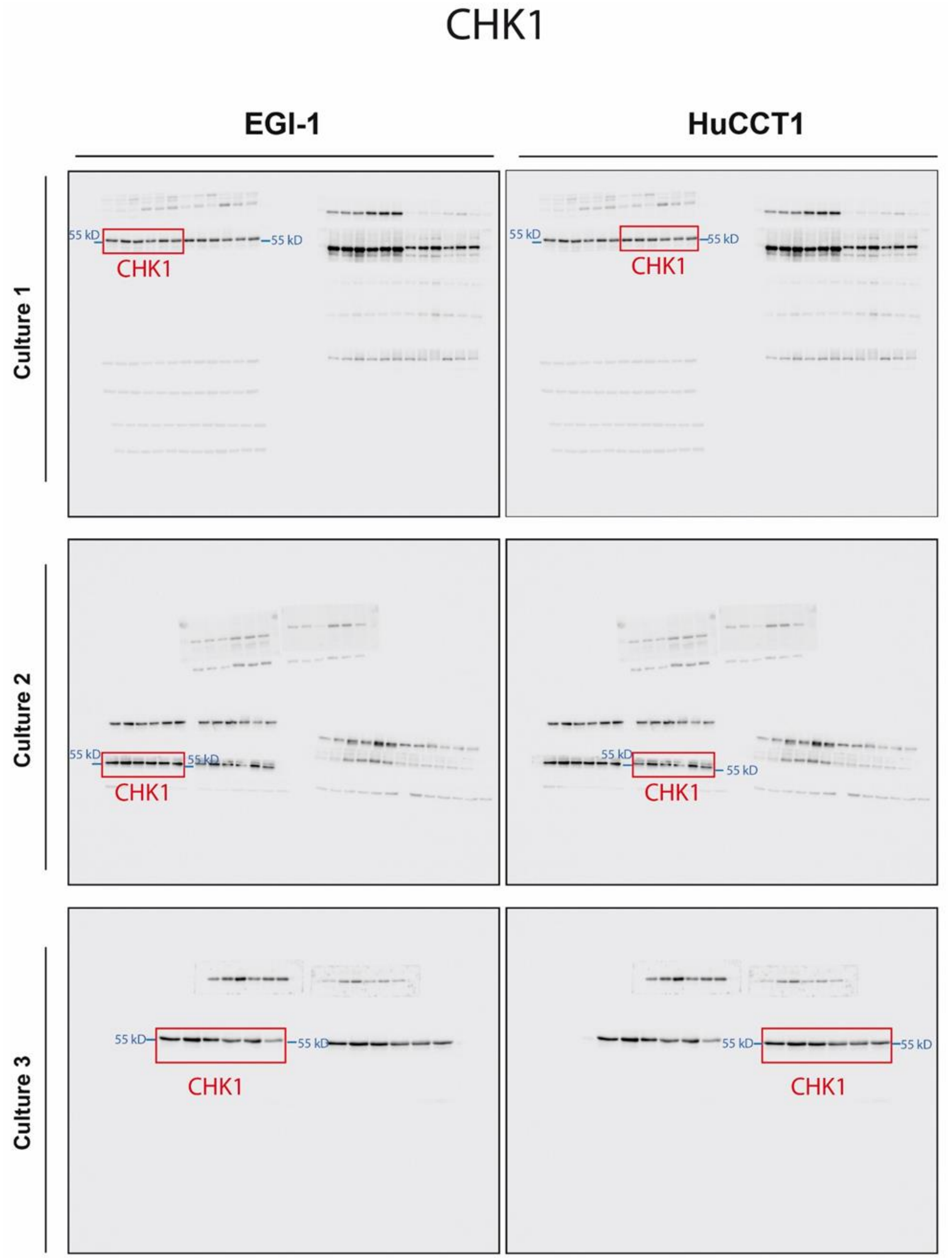

Figure S8.

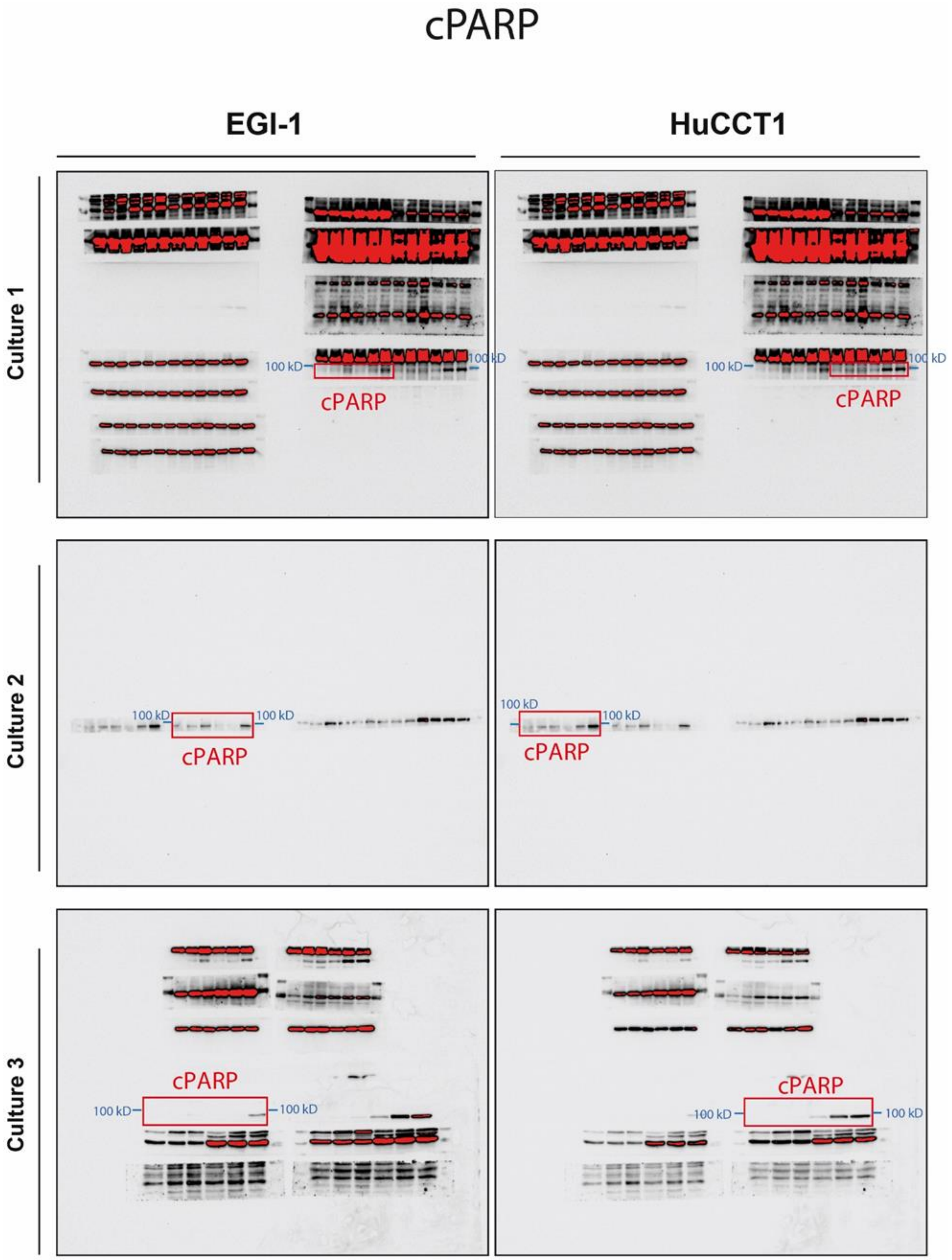

Figure S9.

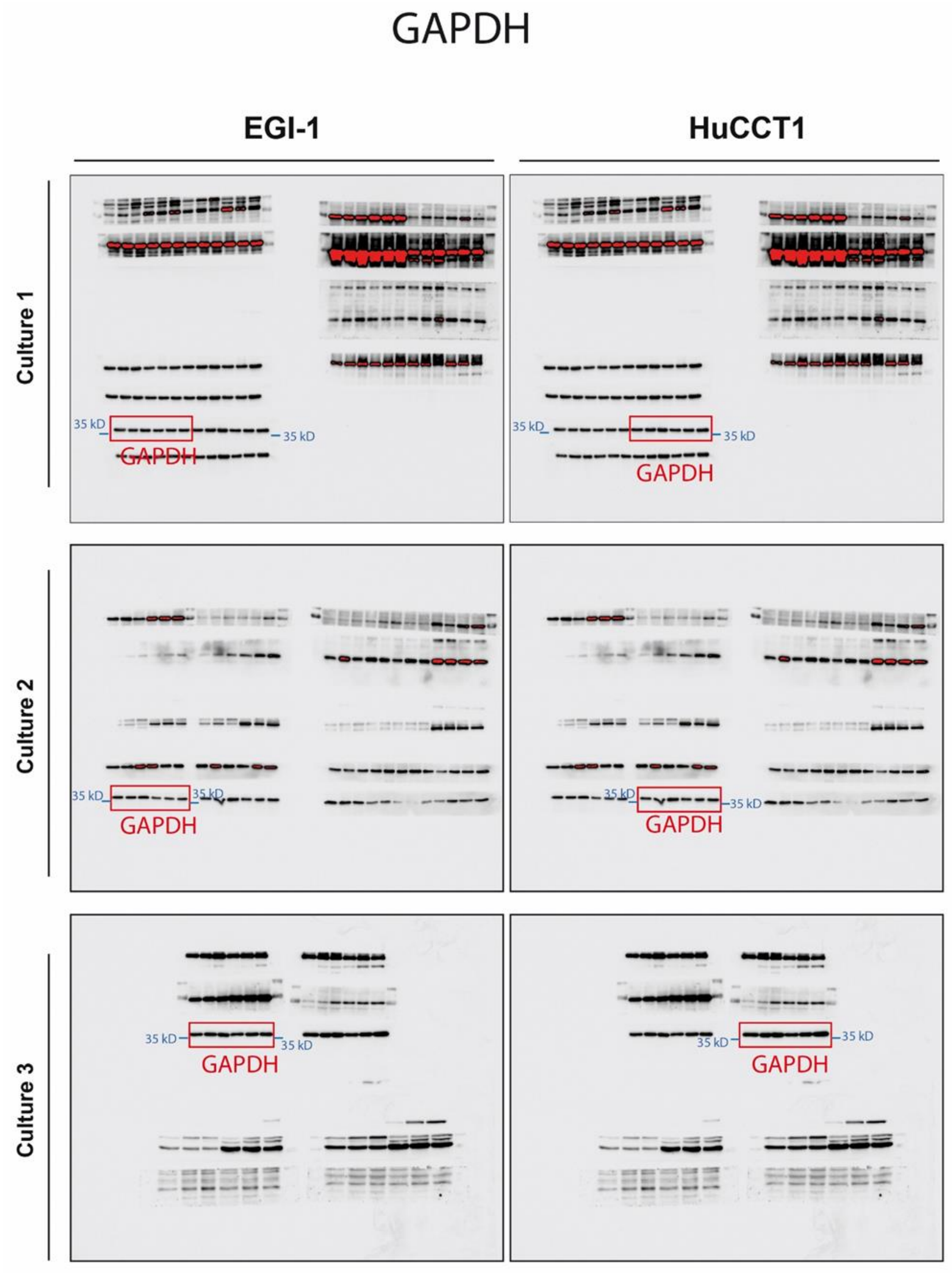

Figure S10.

In the following pages we show Figure S11–S15 that contain the western blots shown in Figure S2, that were performed in human primary isolated hepatocytes, EGI-1 and HuCCT1 cholangiocarcinoma (CCA) cells exposed to plasma activated medium (PAM) or gemcitabine

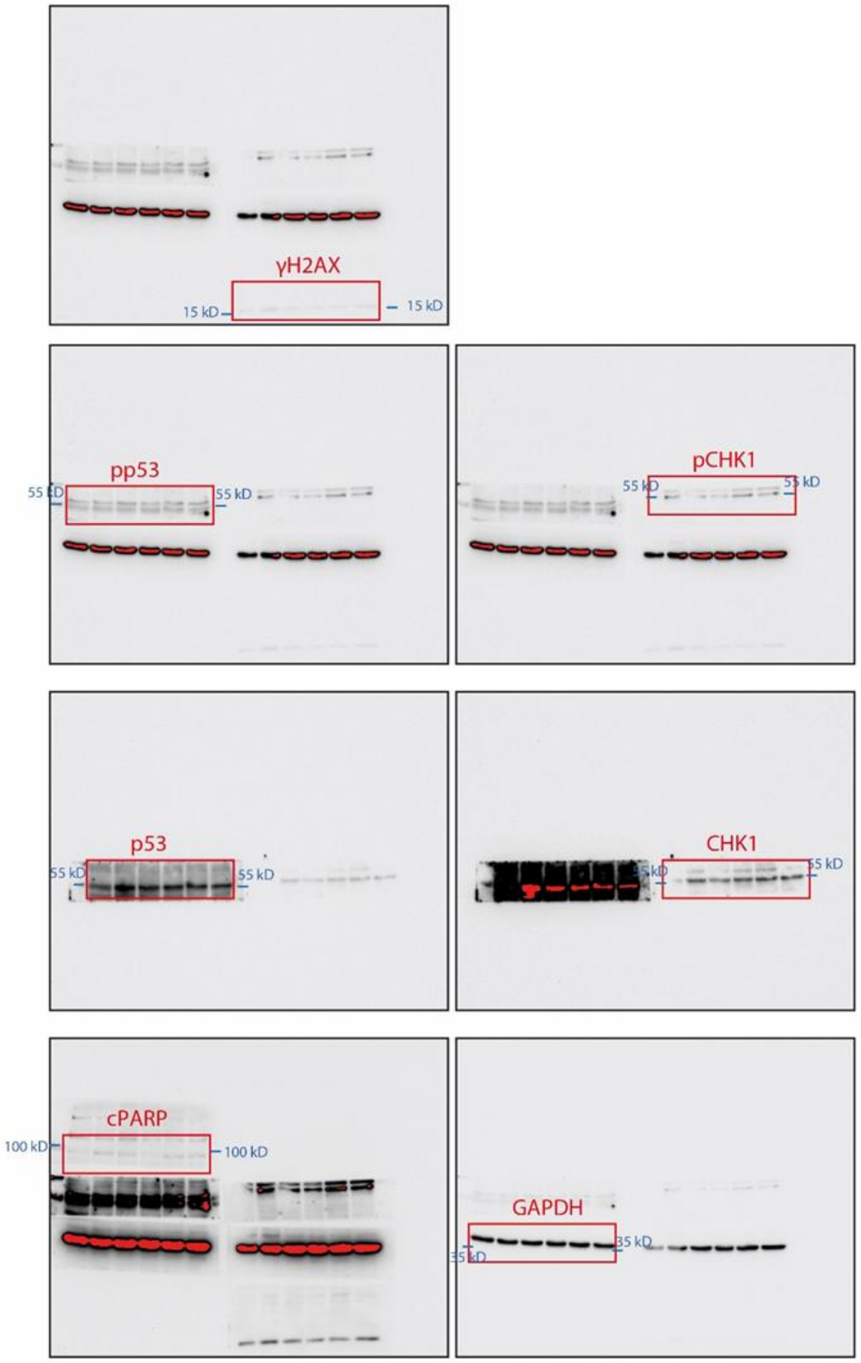

Figure S11. Full western blots corresponding to Figures S2a.

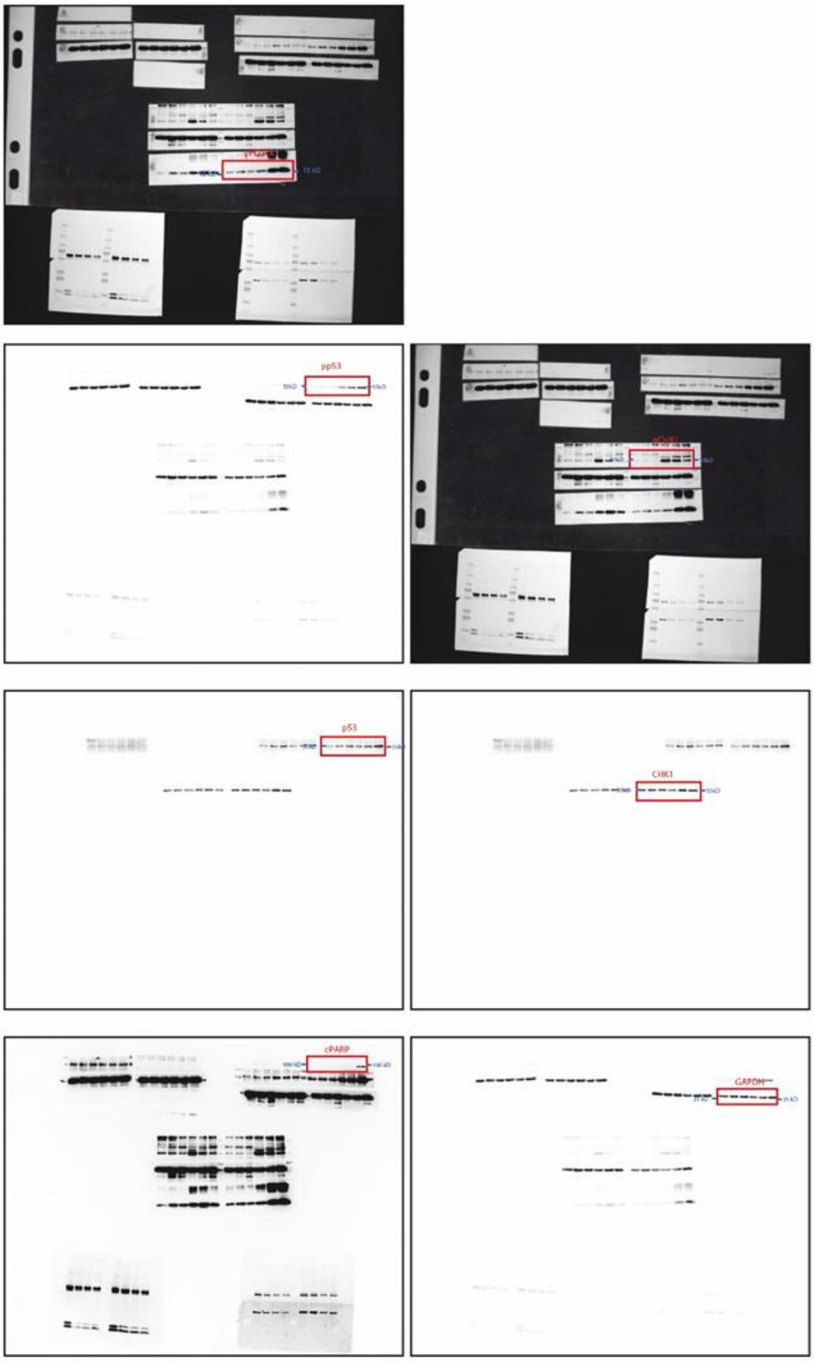

Figure S12. Full western blots corresponding to Figures S2b—EGI-1.

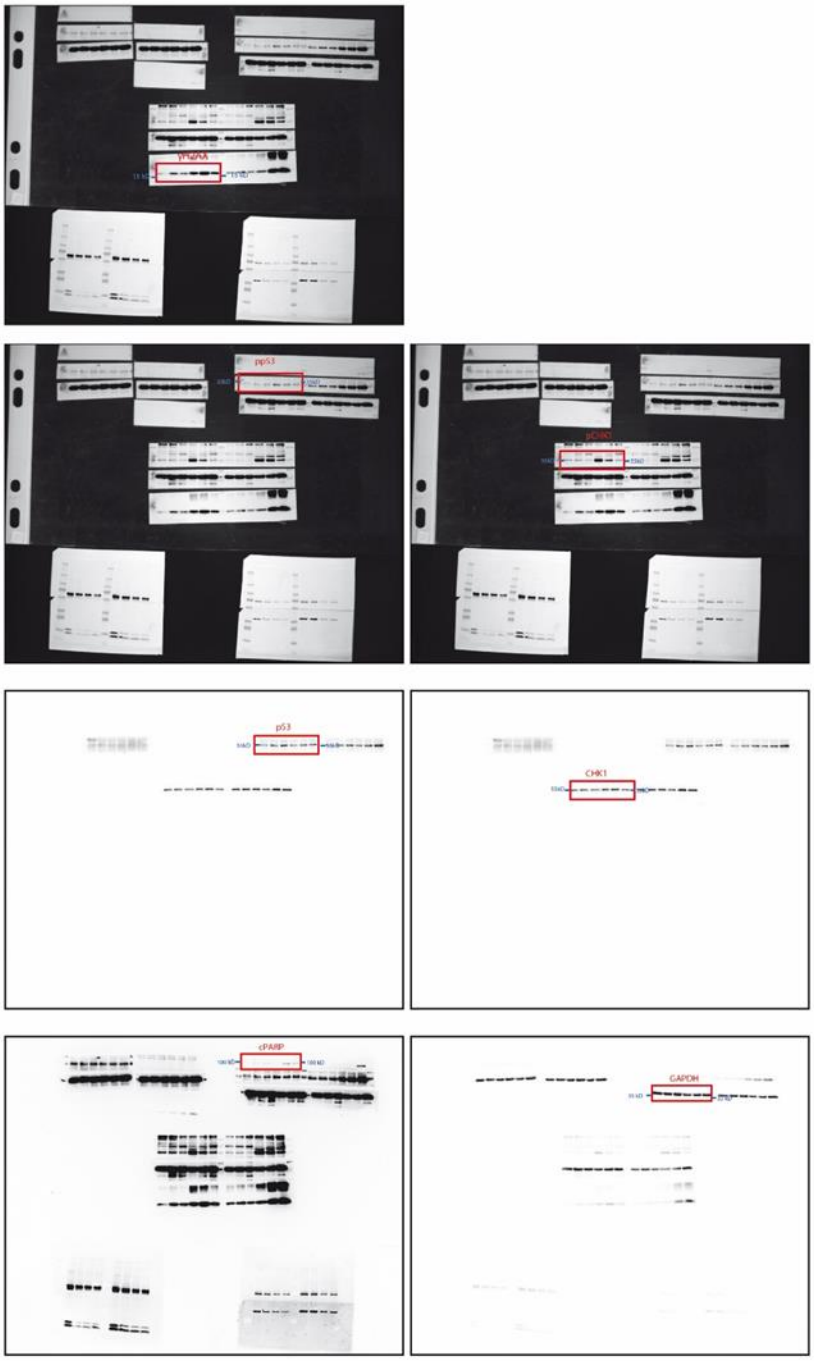

Figure S13. Full western blots corresponding to Figures S2b—HuCCT1.

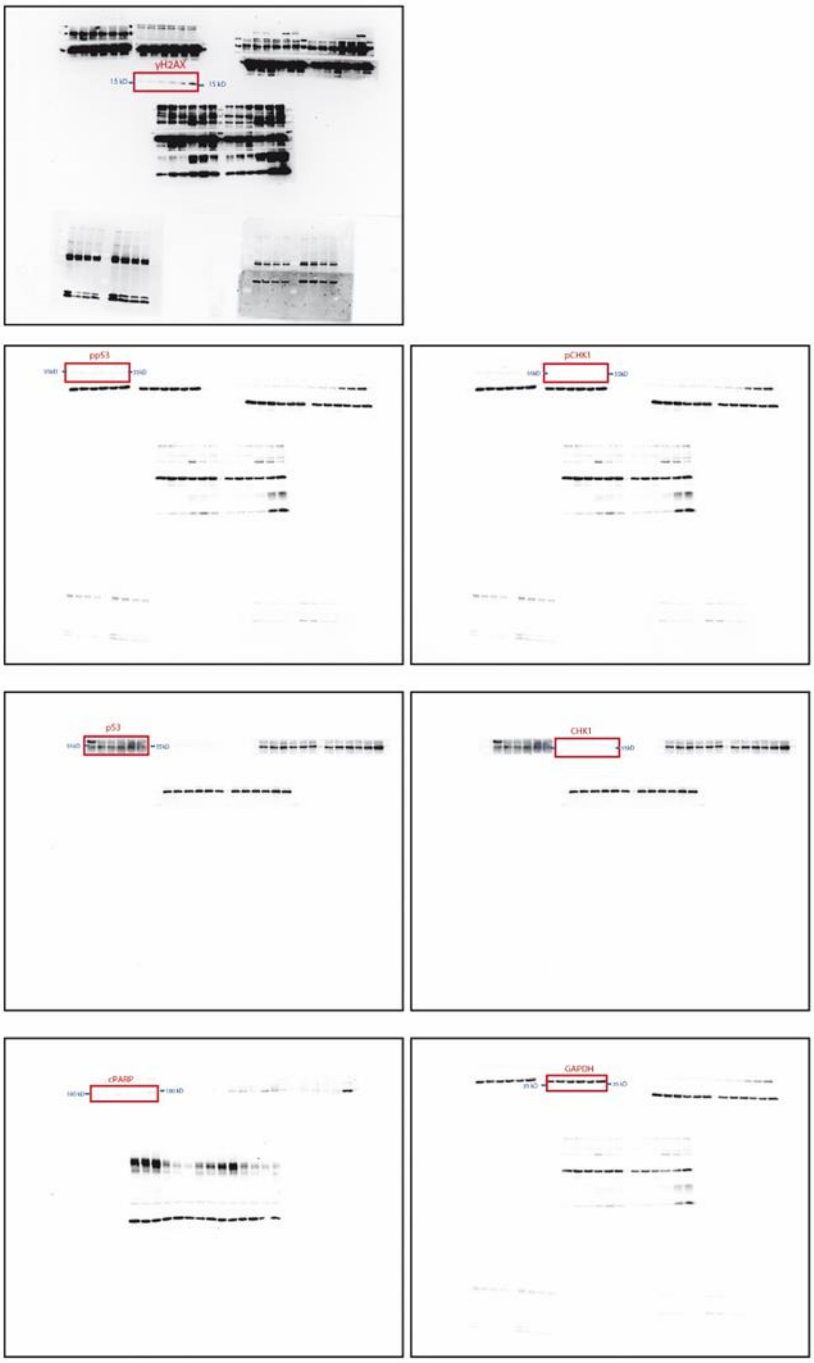

Figure S14. Full western blots corresponding to Figures S2c.

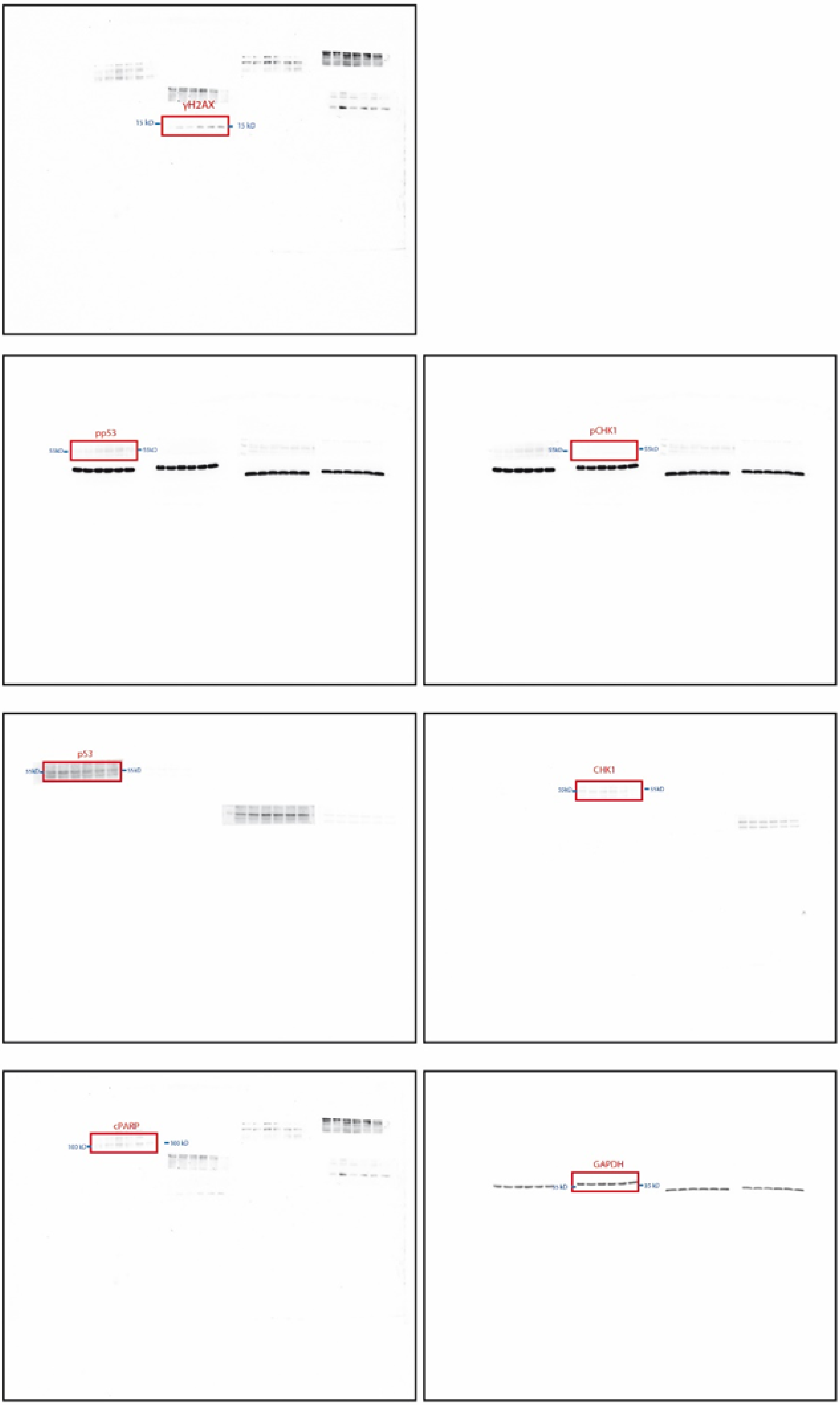

Figure S15. Full western blots corresponding to Figures S2d.

© 2020 by the authors. Licensee MDPI, Basel, Switzerland. This article is an open access article distributed under the terms and conditions of the Creative Commons Attribution (CC BY) license (http://creativecommons.org/licenses/by/4.0/).
